# The Work of Texas Surgeons

**Published:** 1887-04

**Authors:** 


### THE WORK OF TEXAS SURGEONS.

AS SHOWN BY DR. CUPPLES' ADMIRABLE COMPILATION.

What member of the Medical profession in Texas, especially, of the State Medical Association, does not feel an emotion of pride,—exultation we may say,—animate his bosom, when he reads the encomiums of the Medical Press of America on the "Report of Surgery," by the Committee from the Texas State Medical Association? The gratification is the more marked, when we recall the slurs and sarcasms cast upon the profession of this State, up to within a very recent period. Who has not been stung, and felt a sense of indignation at the *Lancet's* unjust remark,—now so familiar, as to be threadbare, "*What good can come out of Texas?*"

The love of approbation is strong in man's nature. We have held that pride,—pride of attainment, of character, of success,—of duty well performed—is not only not a weakness, but is commendable. It is pride that makes the soldier "seek the bubble reputation,"—that makes some men pay their debts, makes others have a decent regard for public sentiment, and stimulates all to noble deeds! The chaste and able *New Orleans Medical and Surgical Journal* says the Report on Surgery by the Committee of the Texas State Medical Association is "something for a whole State to be proud of!"—and we are proud of it, and proud that the profession of Texas, so long belittled—because of *inaction*, has now, by force of its scientific ability, as set forth in this compilation, virtually wrung from the world a recognition, and a place in the ranks of advancing Medical Science; an acknowledgement that they are the peers of the proudest in the land. But for organization,—the watchword of the day,—and of the JOURNAL, we would doubtless be to-day still unknown, unrecognized—nobodies! All honor to our most worthy brother!—This work is a fitting monument to his memory—the crowning jewel of his useful, eventful life.

In another place we collate a few of the criticisms of the press, on the Report of Surgery.

